# Shock in Its Relation to Anesthesia

**Published:** 1908-01

**Authors:** Emory Lanphear

**Affiliations:** St. Louis, Mo., Professor of Surgery in the Hippocratean College of Medicine


For the Texas Medical Journal.

### Shock in Its Relation to Anesthesia.

BY EMORY LANPHEAR, M. D., PH. D., LL. D., ST. LOUIS, MO.,
Professor of Surgery in the Hippocratean College of Medicine.

Much of that which we have been pleased to call "shock" in the past is now recognized as supersaturation with ether or chloroform in some cases, but more particularly poisoning from retained carbon dioxide in most—either alone or with too much anesthetic vapor. This is particularly demonstrated by the effects of hyoscine-morphine anesthesia (used hypodermatically) shock, except that due to profound loss of blood, has been practically eliminated from the work of those who use this method.

The terms "surgical syncope," surgical "shock" and "collapse" have been used rather vaguely and indefinitely by authors in the past. Recent investigations show that three different conditions are included under the term

SHOCK.

(1) Circulatory shock, (2) respiratory shock, (3) nervous shock; to which may well be added (4) composite shock—a mixture of two or all three of the preceding.

1. *Circulatory Shock.*—Syncope may be due to so great loss of blood as to render the brain profoundly anemic; in which case death may occur before the patient can be revived (as in great hemorrhage in trauma, or by serious, continued loss of blood during operative work). Or it may depend upon vaso-motor disturbances of such degree as to temporarily menace life, caused by the character of injury or surgical procedure; belonging, therefore, to the group of "nervous shock," although the immediate symptoms are caused by cerebral anemia.

Circulatory shock, as seen upon the operating table, is about as follows: The patient has been doing well, apparently, in spite of perhaps enormous loss of blood; suddenly there is marked pallor of face and lips, a feeble or imperceptible pulse, cold and sometimes clammy extremities, dilatation of pupils, shallow respiration—general collapse. Here the trouble is not weakness of the cardiac muscle (hence strychnine in circulatory shock is worse than useless), but insufficiency of blood in the heart chambers; there is not enough blood to permit the requisite amount to be sent to the brain. Unless something can be done to quickly restore cerebral circulation death from acute anemia of the brain will follow.

It is in this class of cases that greatest benefit is to be derived from hypodermoclysis, or in condition of great peril from intravenous injection of normal salt solution or even transfusion. As a matter of fact, injection of salt solution for shock is strictly indicated only in this class of cases; hypodermoclysis as well as hypodermatic injection of strychnine being greatly overdone—used injudiciously, if not harmfully—in every part of the world. Preceding hypodermoclysis the head of the table should be lowered and in extremely bad cases the legs bandaged in order to throw as much blood into the brain as possible; and after return of the patient to bed the foot of the bed must be kept elevated until good circulation is restored.

The same kind of syncope sometimes occurs by sudden change of posture, as when a patient in full anesthesia is raised to the sitting position for adjustment of dressings; but is then purely transitory. Of much more serious character is the "shock" which sometimes follows removal of a huge intra-abdominal growth (typically in tumors of the kidney, or other retroperitoneal tumors), which owes its origin to wide dilatation of the splanchnic area, because we do not now possess any agent which can completely and quickly drive the blood from the huge vessels of the abdomen into the brain and skin. Here atropine and glonoin are the indicated drugs, and instant application of heat to the extremities is the next best thing. Lowering the head is also indicated—the exaggerated Trendelenberg position being advisable for some time, with only gradual restoration to the horizontal.

2. *Respiratory Shock.*—The syncope of asphyxia, the supersaturation of the patient with an inhalant anesthetic, the "shock" which often comes on unexpectedly because there has been no great loss of blood, presents much the same symptoms as the circulatory variety, the cyanosis giving way to pallor with dilated pupils and shallow or absent respiration; but the pulse is generally good and in some cases even full and bounding. For here the right heart is embarrassed by distension and labors energetically, tumultuously, to force the blood into the lungs for oxygnation—this continuing until the heart muscle becomes poisoned with asphyxial blood. Most deaths, on the table, from chloroform occur in this way, although it is true, as said by Hewitt, that "there is good evidence that when certain anesthetics are administered in large doses the heart muscle may be suddenly paralyzed, but it is highly probable that when anesthetics are thus administered other important factors capable of bringing about circulatory failure come into play."

Based upon the two statements just made, two rules should be formulated and well fixed in the mind of every anesthetist: (1) The administration of any anesthetic should be begun with extreme care, slowly, with close observation of its effect on pulse; but after anesthesia is well under way the pulse is of only minor import. (2) After the patient is anesthetized the respiration must be the chief guide to safety—any tendency to failure of respiratory effort and particularly any indication to cyanosis must be regarded as a signal of danger and immediately remedied. To wait until there is total abolition of respiration, necessitating cessation of operative procedures and resort to artificial respiration, is, in the vast majority of cases, pure carelessness on the part of the anesthetizer and not "idiosyncrasy" on the part of the patient.

Only too often asphyxia comes from dropping of the tongue into the pharynx or letting the palate close the air passage by permitting the jaw to drop back or down too far. The anesthetist who under such circumstances catches the tongue with forceps and pulls it forward ought to be kicked from the operating room—all that is necessary to do is to remove the pillow from under the patient's head and to pull the jaw forward and upward by placing the thumb and forefinger behind the angle of the jaw and making traction forward and upward.

In this form of shock the first indication is to restore breathing by resorting to artificial respiration if the natural efforts have ceased. If they are merely shallow and irregular oxygen by inhalation is the surest remedy if at hand. If not—which is generally the case when wanted—galvanism is of use; but this, also, is frequently not ready when needed. So one must usually depend upon medicines. Here injection of a large dose of strychnine is permissible, though heart stimulation is not the special object to be attained but stimulation of the respiratory center—the heart will take care of itself if the blood can be oxygenated. Digitalin, as increasing the action of the left heart, would seemingly be indicated; but it must be recalled that the left heart is empty, the right surcharged with poison-ladened blood which can not be forced through the inactive lung. The inhalation of aromatic spirits of ammonia is sometimes good. In bad cases of respiratory failure forcible dilatation of the sphincter ani will start respiration. Hypodermic injection of sulphuric ether or of whisky sometimes stimulates the respiratory center into activity.

As soon as the "shock" has subsided (and it frequently disappears almost as suddenly as it comes on) the operation may be completed, under just as little anesthetic as possible, and the patient put in bed as soon as safety will permit; for these are the cases in which so-called "secondary shock" (not due to congealed hemorrhage) is likely to appear.

3. *Nervous Shock.*—This form of shock is best illustrated by the man whose leg is run over by a locomotive; the loss of blood is not great, the mere observance of a limb should not ordinarily prove fatal, the pain though sudden and excruciating is not greater than often borne, yet the man dies of "shock"—the impress upon the central nervous system has been so great that "reaction" can not take place. Now this exact condition may arise, but fortunately in less degree, during an operation under anesthesia—the "stimulation of the cardio-inhibitory center by afferent impulses through the vagus" being the usual explanation. But, whatever the mode of conduction, by reason of tremendous disturbance of the vaso-motor system this form of shock is greatly to be feared; and there can be no doubt that the pernicious influence of the anesthetic itself, in too large quantity, upon the nerve centers has much to do with its production. The lesson of the anesthetist is, therefore, to learn how to carry the patient up to the critical period of operation with the smallest possible quantity of anesthetic, whatever it may be, yet have the anesthesia so complete at the proper point that a severance of the great nerve trunks shall not be felt, or that ligation of any huge vessel (like the femoral or subclavian) shall not be attended by any disturbance sufficient to jeopardize life. This is difficult to do and is one of the things which makes the experien[illegible] surgeon often say that the responsibility of the anesth[illegible]equently greater than that of operator.

[illegible]tment of this form of shock strychnine finds its proper use: 4 milligrams (1-15th of a grain) may be injected [illegible] soon as the shock is apparent. Glonoin, too, is often of advantage in dose of one milligram (gr. 1-67th). Hypodermoclysis is of doubtful utility, though commonly used. This form of shock is almost unknown when the hyoscine-morphine-cactin anesthesia is used—one of the great advantages of this combination.

4. Secondary shock is very often merely the effects of too much anesthetic, plus more or less carbon-dioxide poisoning from replacement of pure air by excess of the vapor of ether or chloroform for a long time. It is particularly apt to occur phen large quantities of ether have been given by a man who believes in "crowding it on" until the patient is cyanotic.

This asphyxiation or carbon-dioxide poisoning, added, to the direct effect upon excretions and metabolism of the anesthetic itself, constitutes the fourth or "composite" variety of "shock" during and after anesthesia.

Its treatment is essentially that of the effects of asphyxia: plenty of fresh air, elimination by the skin (induced by hypodermoclysis if necessary, or by subcutaneous use of pilocarpine); washing out the stomach, in which large quantities of ether (and in less degree chloroform) may be found; free catharsis by giving a large dose of Epsom salt in the stomach at the conclusion of lavage, or by hypodermic injection of salicylate of physostigmine, 1 milligram (1-60th grain) every hour four times, and by use of high enemas containing ox gall and turpentine, and especially by the external application of heat.

---

Dr. C. A. Smith, Texarkana, Chief Surgeon Cotton Belt line, we are glad to note, was recently elected President of the Tri-State Medical Society—Texas-Louisiana-Arkansas.

